# Characteristics and Outcomes of Patients with Partial Do Not Resuscitate Orders in a Large Community Hospital

**DOI:** 10.7759/cureus.6048

**Published:** 2019-11-01

**Authors:** Ali Rafiq, Waqas Ullah, Mary Naglak, Doron Schneider

**Affiliations:** 1 Internal Medicine, Abington Hospital, Jefferson Health, Abington, USA

**Keywords:** resuscitation, partial code, medical ethics, palliative care, end-of-life care, rapid response team, community hospital, partial do not resuscitate (dnr)

## Abstract

Background

Formal writing of do not resuscitate (DNR) orders first appeared in the literature in the late 20^th^ century. Recently, providers have also noticed the presence of partial DNR orders while caring for patients. We sought to determine the effect of these orders on the clinical outcomes of the patients.

Methods

The study was a retrospective chart review covering a period of approximately 30 months. Patients included in the study were over 18 years of age and had a partial DNR order (i.e., chemical code, do not defibrillate (DND), do not intubate (DNI), intubate only, no cardiopulmonary resuscitation (CPR)) entered during hospitalization. Primary medical problems were categorized by organ system and the outcome was stated in terms of their disposition and mortality.

Results

A total of 71,143 code orders were entered during the study period, with partial DNR orders accounting for 1.8% of these orders (chemical code 2%, DND 0.8%, DNI 48%, intubate only 38%, and no CPR 10%). About 38% of all patients were discharged to home, 32% were discharged to a facility, and 11% were discharged on hospice. More than half of the patients did not have a palliative care consult. Of all the patients having partial code orders, about 150 patients had a rapid response team called on them and five patients had a cardiac arrest with a code blue activated on them. The mortality of these patients was significantly higher than other patients possibly due to confusing code orders. Surprisingly, a higher percentage of patients (19%) with a mean age significantly lower (p < 0.001) than discharged patients had inpatient mortality.

Conclusion

Our study demonstrates the first reported prevalence of partial DNR orders in the general inpatient population and its possible detrimental effects on the patient clinical course. This study offers several opportunities for quality improvement, such as developing prompts for the healthcare team to involve palliative care services more often for such patients.

## Introduction

It is common practice to ask a patient’s “code status” upon admission to the hospital. This question essentially means that you are inquiring about a patient’s wishes in the event that he/she reaches a point of clinical deterioration that necessitates cardiopulmonary resuscitation (CPR). A do not resuscitate (DNR) order is meant to imply that a patient would not wish resuscitation in case of cardiopulmonary arrest. Formal writing of DNR orders first appeared in the literature in the early 1980s [1]. More recently, however, orders for partial DNRs have been incorporated into clinical practice [2], raising ethical and management queries. Partial DNRs can be of several forms, with the following types seen commonly in daily practice:

□ Intubate only, also known as a “cardiac DNR”, whereby the patient is agreeable to being intubated but does not wish chest compressions in the event of a cardiopulmonary arrest

□ Do not intubate (DNI), whereby the patient is agreeable to chest compressions but does not wish to be intubated in the event of a cardiopulmonary arrest

□ Chemical code, whereby the patient is agreeable to the administration of medications but is not agreeable to chest compressions or intubation in the event of a cardiopulmonary arrest

□ Do no defibrillate (DND), whereby the patient is agreeable to being resuscitated with chest compressions and intubation but does not want to be defibrillated

A fifth type of partial DNR also exists, known as a “show code”, whereby the healthcare team indulges in an action or set of actions to psychologically appease the patient’s family with the knowledge that such an action will have no benefit [3]. The reasoning behind such intervention is not usually strong enough to perform the intervention and hence appears to be ethically problematic [4]. Additionally, a sixth type of partial DNR order has been described that presents itself as a checklist for patients. This checklist appears as a “menu,” offering patients and caretakers a variety of options to choose from in the event of cardiopulmonary arrest [5]. DNR orders, in general, can be hard to comprehend for patients and families; hence, such an approach promises only to confuse them even more.

Due to the ethical complexities attached with a partial DNR order, its use has been discouraged in an attempt to establish accurate and safe treatment plans [4]. Other authors have described this order as “confusing” [3], whereas at least one community hospital has been reported to forbid its use altogether [6].

Studies looking at the outcome of patients admitted to a hospital with partial DNR orders are scarce. In 2001, Dumot et al. demonstrated that the survival of patients with limited (or partial) DNR orders who suffered from a cardiopulmonary arrest in the hospital was zero percent [7]. However, the general characteristics and outcomes of these patients are poorly known. Based on our literature review, there is also no data on the outcomes of these patients when they are approached by a rapid response team (RRT). We sought to determine the outcomes of patients having partial code orders in terms of their inpatient mortality and also their clinical course, especially in a situation where an RRT was called for their worsening conditions. This study provides the burden of these partial code orders and unmasks its implications on the care of patients.

## Materials and methods

This was a retrospective chart review observational cohort study performed at Abington Hospital - Jefferson Health, Abington, PA. Data for all patients admitted between June 15, 2016 and January 10, 2019 were extracted. The study was approved by the institutional review board at Abington Hospital. The inclusion criteria were age 18 years and above and patients with the following code orders: chemical code, do not defibrillate (DND), do not intubate (DNI), intubate only, and no CPR. Patients aged less than 18 years and those admitted to the inpatient rehabilitation unit were excluded. Additionally, patients with the following orders were also excluded: full code order, comfort measures only, DNR/DNI, and DNR/no code.

Data collection

The data were collected in three phases. Phase 1 included extracted data for all code orders entered within the study period. Phase 2 included extracted data based on inclusion criteria and exclusion criteria as mentioned above. Meanwhile, Phase 3 included extracted data based on age restrictions, and duplicate visits were omitted. For example, if two partial DNR orders were entered in the same admission, the latter entry was deleted.

Data analysis

Data analysis for this study was conducted in three phases. Phase 1 included the total number of code orders entered during the study period. Phase 2 included the frequency of various partial DNR orders as described in the inclusion criteria and the frequency of decision-makers involved was determined. Phase 3 included descriptive statistics including mean, median, standard deviation, and interquartile range were determined for all continuous variables (age, length of stay, midnights spent in the critical care unit, and the number of times RRT was called). Frequencies and percentages were calculated for all categorical variables (gender, presence of dementia, primary medical problem, the involvement of the resident team at the time of admission, and outcome).

An “outcome” was defined as an event that leads to the termination of hospitalization. The following outcomes were noted and recorded:

□ Discharge to home

□ Discharge to a facility (skilled nursing facility, inpatient rehabilitation facility, inpatient psychiatric facility, long-term acute care hospital, or transfer to other hospitals)

□ Discharge to hospice (home or inpatient)

□ Death

A “primary medical problem” was defined as the most clinically pertinent medical condition that was treated during a hospitalization. The primary source of this information was the “principal diagnosis” coded at the end of the hospitalization. If the “principal diagnosis” was absent or nonspecific, the “discharge diagnosis” was recorded. If the “discharge diagnosis” was absent or nonspecific, the primary medical diagnosis mentioned in the discharge summary was used. For the sake of simplicity, primary medical problems were categorized into organ systems, with sepsis and cancer-related diagnoses being grouped separately.

Continuous variables were assessed for normality; several variables were skewed (length of stay, midnights spent in the critical care unit, and the number of times RRT was called), and thus, a square root transformation was performed. For normally distributed continuous variables (age) and variables successfully transformed (length of stay, midnights in the critical care unit, and the number of times the RRT was called), comparisons between different outcomes were made using analysis of variance. Chi-square analysis was used to determine associations between different outcomes and various categorical variables.

All data were analyzed using the IBM Statistical Package for Social Sciences (SPSS) for Windows, Version 20.0 (IBM SPSS Statistics, Armonk, NY). All p-values were two-tailed, and a level of < 0.05 was considered significant.

## Results

**Figure 1:**
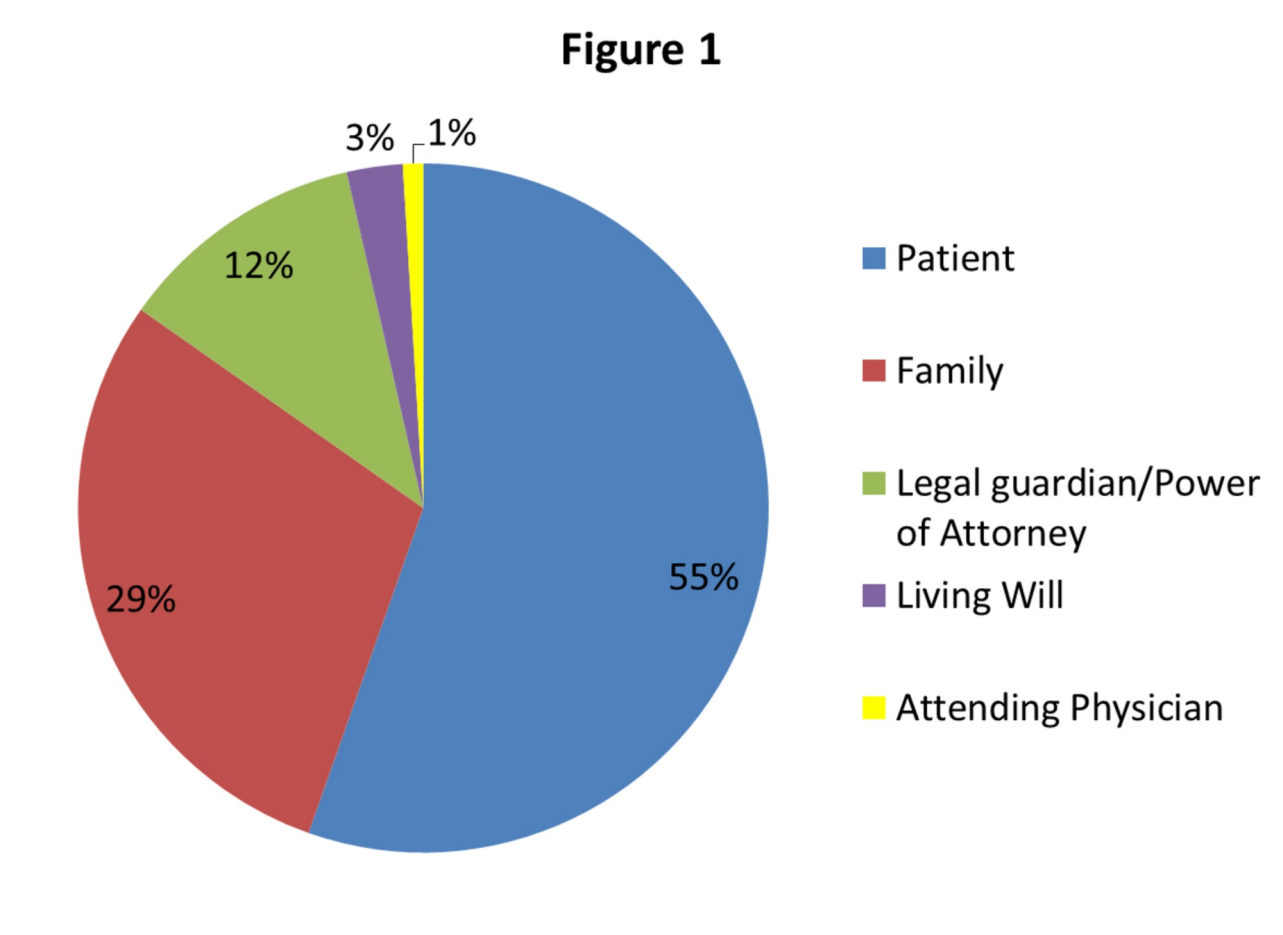
Decision maker for partial Do Not Resuscitate order

During the 30 month study period, 71,143 code orders were entered of which 1,260 (1.77%) were identified as partial DNR orders. The frequencies of the different types of partial DNR orders were: chemical code - 26 (2%), DND - 10 (0.79%), DNI - 609 (47.62%), intubate only - 484 (38.41%), and no CPR - 131 (10.40%). These orders were entered based on various sources with the vast majority based on patient and family requests (Figure 1).

After omitting data points as outlined in the Materials and Methods section above (Phase 3), 1,150 entries were included. Of the study population, 55.8% were female with the median age being 82 years (mean: 78.9 ± 12.7 yrs.). The median length of stay was 5 (mean: 7.9 ± 9.7) midnights, whereas the median number of midnights spent in the critical care unit was 0 (mean: 2.8 ± 5.9). Additionally, 13.7% had a history of dementia, and palliative care services were consulted in 35.6% cases. Resident teams were involved at the time of admission in 92.5% of cases.

Further analysis revealed that 38.5% of patients were discharged to home, 31.7% were discharged to a facility, 19% died, and 10.7% were discharged to hospice. Organ systems of the primary medical problems and their respective frequencies in various outcomes are provided in Table 1.

**Table 1:** Organ Systems of Primary Medical Problems and Respective Frequencies in Various Outcomes

Primary Medical Problem	Home	Death	Hospice	Facility	Total (n)
Pulmonary	80	38	22	60	200
Sepsis	65	40	25	55	185
Cardiovascular	58	39	17	51	165
Neurological	61	36	12	50	159
Gastrointestinal	40	15	12	36	103
Renal	30	11	11	22	74
Cancer-related diagnosis	23	6	8	17	54
Musculoskeletal	22	9	5	18	54
Hematological	8	9	2	5	24
Endocrine	6	3	0	2	11
Rheumatologic	1	1	0	1	3
Other	49	12	9	48	118
					1,150

Outcomes were not affected by the involvement of palliative care services or having a history of dementia. Inpatient mortality was significantly higher in males (26.2%) than in females (13.4%) (p < 0.001). Additionally, the mean age for inpatient mortality (74.4 ± 13.4 years) was significantly (p < 0.001) lower than that of patients discharged to home (77.9 ± 13.1 years), hospice (80.2 ± 12.3 years), or a facility (82.6 ± 10.6 years).

Furthermore, the mean length of stay (in midnights) was significantly (p < 0.001) lower in patients discharged home (4.5 ± 6.1, median: 3) when compared to patients discharged to a facility (9.7 ± 10.3, median: 7), to hospice (8.6 ±7.1, median: 7), and patients who died (11.2 ± 13.2, median: 7). Additionally, 42.9% of patients who died spent at least 10 midnights in the hospital.

Amongst patients discharged to a facility, the mean number of midnights spent in the critical care unit (2 ± 4.8, median: 0) was significantly lower than patients discharged home (2.9 ± 5.5, median: 0) (p = .027) and patients who died (3.5 ± 7.4, median: 0) (p = .019). There was no significant difference between the patients who were discharged home and those who died (p = .965).

Additionally, 13% of patients had at least one RRT called on them. Amongst this group, 12% were discharged home, 33% were discharged to a facility, and 18% were discharged to hospice. The inpatient mortality among these patients was 38% which was strikingly higher than the patients having partial code orders or no activation of RRT. 

Five patients suffered from cardiac arrest while partial DNR orders were still active. Return of spontaneous circulation (ROSC) was not achieved in any of these cases. These patients had a 100% mortality irrespective of their age, floor, or nature of the underlying disease. Additional details regarding these patients are provided in Table 2.

**Table 2:** Patients Suffering from Cardiac Arrest with Active Partial DNR Order DNI: do not intubate; DNR: do not resuscitate; GI: gastrointestinal; GMF: general medical floor; MICU: medical intensive care unit; PEA: pulseless electrical activity, PC: palliative care; POA: power of attorney; Vfib: ventricular fibrillation; Vtach: ventricular tachycardia

	Case 1	Case 2	Case 3	Case 4	Case 5
Partial DNR Order	DNI	Intubate only	Intubate only	DNI	Chemical code
Age (years)	81	88	69	73	74
Primary diagnosis	Intestinal obstruction	Heart Failure	Multiple myeloma	Lower GI hemorrhage	Acute hypoxic respiratory failure
Source	POA	Family	Family	Patient	Family
Floor	MICU	Telemetry	GMF	GMF	MICU
Shift	Night	Night	Night	Night	Day
Dementia	Yes	No	No	No	Yes
PC involvement	Yes	No	No	No	Yes
Documented rhythm	PEA	None	Asystole	Asystole	PEA/Vfib/Vtach

## Discussion

Based on our literature review, our results are the first reported prevalence of partial DNR orders in the general inpatient population. Additionally, partial DNR orders are more prevalent in the geriatric population. This finding is similar to a systematic review and meta-analysis published by Cook et al. done on DNR orders, which showed DNR orders had an adjusted odds ratio of 1.70 and 2.96 in patients aged 75 to 84 and ≥ 85 years, respectively, when compared to patients less than age 65 years [8]. The prevalence of dementia in our study population (13.7%) was also similar to previously reported studies, particularly in individuals aged 71 and older (13.9%) [9].

Inpatient mortality, however, was more prevalent in younger patients within the studied population. Mortality was also associated with a longer mean length of stay in our study. This was similar to a finding in a recent study that showed patients in the upper quartile length of stay (LOS) had higher odds of mortality (odds ratio = 1.45, 95% confidence interval (CI) 1.43 - 1.47) than those in the lowest quartile [10]. In our study, overall, more than half of the patients were not discharged home, and nearly one-third either died in the hospital or were expected to die within the next six months (criteria for discharge on hospice).

Sepsis was the most common cause of death, followed closely by cardiovascular, pulmonary, and neurological causes. The overall sepsis mortality in the studied population was 21.6%, which was higher than the state in-hospital mortality rate of 10.5% reported in 2016 [11]. Additionally, sepsis accounted for 18.3% of the total inpatient mortality in our study, whereas the same number was 39.7% in the above-mentioned report [11].

It is not uncommon for patients to depend on family or other surrogates to make decisions for them, particularly in the critical care unit [12]. Our study highlighted that partial DNR orders were requested by the patient's family, legal guardian, or power of attorney in up to 41% of the cases. Furthermore, the role of palliative care is increasingly being recognized as a key component of the critically ill patient’s care plan [13], with about 90% of United States (US) hospitals with more than 300 beds reportedly having an inpatient palliative care team [14]. This service was utilized in less than half the cases in our study population.

Amongst patients who suffered from a cardiac arrest with a partial DNR order active, our findings were similar to the study by Dumot et al. [7], with none of the patients surviving until discharge. The remaining patients who died either were changed to full code or were made DNR/DNI later in their hospital course.

Based on our findings, we put forth certain recommendations. First, hospitals should simplify code orders. At our institution, for example, two partial DNR orders - “Intubate only” and “No CPR” - are similar. Hence, they should be combined for the sake of simplification. Also, the “DNR/No code” order can be confusing for providers, since some might interpret it as “No CPR”, particularly in the presence of an alternate “DNR/DNI” order. Therefore, we recommend the former order be removed altogether. Additionally, the “chemical code” order should be removed since its application is unclear and it has questionable usefulness. The “Do not defibrillate” order should also be removed since shockable rhythms are associated with superior outcomes [15-16].

For patients surviving hospitalization that are not discharged on hospice and do not have advanced directives, we encourage communication with the patients' primary care providers to have goals of care discussions in subsequent follow-up visits since such discussions within the hospital setting are infrequent and tend to occur too late [17]. In 2001, Tierney et al. demonstrated that discussions regarding advanced directives led to increased patient satisfaction in the outpatient setting, particularly in the elderly population [18]. Furthermore, advance directives can lower the cost of healthcare, as suggested by Chambers et al. [19].

Additionally, our study presents an opportunity for increased involvement of palliative care services in patients with partial DNR orders. Hospital systems can look at developing prompts within the electronic health record that would advise providers to consult palliative care when a partial DNR order is placed.

Finally, we propose that hospitals view the presence of a partial DNR order in patients with an RRT called as a possible marker of poor outcome. Our study highlights this finding, with more than half the patients with an RRT called in this study population either dying in the hospital or expected to die within the next six months. At least one previous study has demonstrated relatively poorer outcomes in geriatric RRT patients [20], which echoes our findings since the median age of our study population (82 years) is in the geriatric age group as well.

Our study has several limitations. We did not study baseline patient factors apart from age, gender, and a history of dementia. It would not be surprising if future studies demonstrate that poorer outcomes were associated with an increased frequency of comorbid conditions in this patient population. Additionally, we presented our data based on organ systems, instead of primary diagnoses, apart from sepsis. This was done for the sake of simplification. Further studies are needed to look at outcomes associated with particular diagnoses. Also, we did not stratify our results based on the patients’ primary service. This was particularly difficult to do since many patients changed services during hospitalization.

## Conclusions

Our study revealed increased mortality in patients with partial DNR orders, despite their younger age, and in patients with longer mean lengths of stay. They tended to have even higher mortality if a rapid response team was called due to their worsening condition (38%) or if they had a cardiac arrest (100%). We believe that early palliative care consultation is imperative to decide about the goals of care in such patients.
